# Patient perspectives on digital healthcare technology in care and clinical trials for motor neuron disease: an international survey

**DOI:** 10.1007/s00415-022-11273-x

**Published:** 2022-07-18

**Authors:** Jochem Helleman, Barbara Johnson, Cory Holdom, Esther Hobson, Deirdre Murray, Frederik J. Steyn, Shyuan T. Ngo, Anjali Henders, Madhura B. Lokeshappa, Johanna M. A. Visser-Meily, Leonard H. van den Berg, Orla Hardiman, Anita Beelen, Chris McDermott, Ruben P. A. van Eijk

**Affiliations:** 1 Department of Rehabilitation, Physical Therapy Science and Sports, UMC Utrecht Brain Center, University Medical Center Utrecht, Utrecht, the Netherlands; 2 Center of Excellence for Rehabilitation Medicine, UMC Utrecht Brain Center, University Medical Center Utrecht, and De Hoogstraat Rehabilitation, Utrecht, the Netherlands; 3 Australian Institute for Bioengineering and Nanotechnology, The University of Queensland, Brisbane, Australia; 4 UQ Centre for Clinical Research, The University of Queensland, Brisbane, Australia; 5 Department of Neuroscience, Sheffield Institute for Translational Neuroscience, University of Sheffield, Sheffield, UK; 6 Academic Unit of Neurology, Trinity College Dublin, Dublin, Ireland; 7 Physiotherapy Department, Beaumont Hospital, Dublin, Ireland; 8 School of Biomedical Sciences, Faculty of Medicine, University of Queensland, Brisbane, Australia; 9 The Royal Brisbane and Women’s Hospital, Herston, Australia; 10 Wesley Medical Research, The Wesley Hospital, Auchenflower, Australia; 11 Centre for Clinical Research, The University of Queensland, Brisbane, Australia; 12 Institute for Molecular Biosciences, The University of Queensland, Brisbane, Australia; 13 Department of Neurology, UMC Utrecht Brain Centre, University Medical Centre Utrecht, Heidelberglaan 100, 3584 CX Utrecht, the Netherlands; 14 Department of Neurology, National Neuroscience Centre, Beaumont Hospital, Dublin, Ireland; 15 FutureNeuro SFI Research Centre, Royal College of Surgeons in Ireland, Dublin, Ireland; 16 Biostatistics and Research Support, Julius Center for Health Sciences and Primary Care, University Medical Center Utrecht, Utrecht, the Netherlands

**Keywords:** Motor neuron disease, Amyotrophic lateral sclerosis, Digital technology, Survey, Patient perspective

## Abstract

**Introduction:**

To capture the patient’s attitude toward remote monitoring of motor neuron disease (MND) in care and clinical trials, and their concerns and preferences regarding the use of digital technology.

**Methods:**

We performed an international multi-centre survey study in three MND clinics in The Netherlands, the United Kingdom, and Australia. The survey was co-developed by investigators and patients with MND, and sent to patients by e-mail or postal-mail. The main topics included: patients’ attitude towards remote care, participating in decentralized clinical trials, and preferences for and concerns with digital technology use.

**Results:**

In total, 332 patients with MND participated. A majority of patients indicated they would be happy to self-monitor their health from home (69%), be remotely monitored by a multidisciplinary care team (75%), and would be willing to participate in clinical trials from home (65%). Patients considered respiratory function and muscle strength most valuable for home-monitoring. The majority of patients considered the use of at least three devices/apps (75%) once a week (61%) to be acceptable for home-monitoring. Fifteen percent of patients indicated they would not wish to perform home-measurements; reporting concerns about the burden and distress of home-monitoring, privacy and data security.

**Conclusion:**

Most patients with MND exhibited a positive attitude toward the use of digital technology in both care and clinical trial settings. A subgroup of patients reported concerns with home-monitoring, which should be addressed in order to improve widespread adoption of remote digital technology in clinical MND care.

**Supplementary Information:**

The online version contains supplementary material available at 10.1007/s00415-022-11273-x.

## Introduction

Patients with motor neuron disease (MND) experience progressive muscle weakness due to the deterioration of motor neurons, limiting their ability to communicate and perform daily tasks [1, 2]. Besides physical impairments, about half of patients may also develop cognitive impairment, such as frontotemporal dementia [3]. Eventually the disease leads to death on average in 2 to 4 years as a result of respiratory failure [1, 2]. The rate of disease progression, and the occurrence and severity of symptoms varies greatly among patients. Therefore, a flexible approach towards management is required such that the patient’s clinical condition is monitored at intervals that best reflect both the needs of the patient, and the trajectory of their disease. Moreover, despite the added value of attending multidisciplinary clinics, visits may be perceived by patients as excessively time-consuming, and can be challenging for caregivers, especially when patients experience severe physical disabilities [4, 5].

Remote digital technologies have the potential to reduce the burden, and improve the accessibility and personalization of care and clinical trials by enabling tailored collection of disease-related outcomes from home, and facilitating communication between patients and healthcare professionals [6, 7]. In addition, remote digital technologies can accelerate the search to find effective treatment [8–13]. Despite these clear benefits, the real-world use of remote digital technology in MND has been limited, for a large part due to financial barriers [6, 14]. However, since the COVID-19 pandemic, there has been an increase in the adoption of policies that allow for billing and reimbursement for telehealth, together with an increase in the use of telehealth in MND care [15–23].

To further facilitate the wide-scale adoption and utilization of digital healthcare technologies in MND care and clinical trials, a road map has been recently published [24]. One of the main objectives of the road map is to find a set of reliable digital outcome measures that can be captured by patients with MND from home, through a user-centered co-design approach. This approach includes the involvement of end-users (e.g. patients) throughout the process, so that an innovation fits their needs [25]. To date, there is limited information available regarding patient preferences for digital technology. Understanding the user perspective is essential to achieve long-term adherence and adoption in the community. In this international multi-centre study, therefore, we aim to capture patients’ attitudes toward remote MND care and monitoring from home, together with their preferences and concerns about digital technology. We also evaluate differences in patients’ perspectives between countries.

## Methods

### Study design, population and setting

This cross-sectional, multi-center survey study aimed to include patients aged 18 and over with a diagnosis within the spectrum of motor neuron disease including Amyotrophic Lateral Sclerosis (ALS), Primary Lateral Sclerosis (PLS) and Progressive Muscular Atrophy (PMA), at all stages of disease and irrespective of cognitive impairment. There were no exclusion criteria. Ethics approval was obtained from the local ethics committees prior to the start of the study, and patients provided either written or digital informed consent before participating. All survey data was collected between November 2020 and November 2021. The present study was conducted by multidisciplinary MND clinics in Utrecht, The Netherlands; Sheffield United, Kingdom (UK); and Brisbane, Australia.

### The survey

The survey was developed in English and Dutch by investigators from the participating clinics, in collaboration with eight patients with MND. The main topics included: patients’ preferences for and concerns with performing measurements at home, and patients’ attitude towards receiving care remotely and participating in decentralized clinical trials. Additional topics included the current use of digital technology in daily life and healthcare. Patients answered options on a 5-point Likert scale ranging from ‘Totally disagree’ to ‘Totally agree’, and from ‘Not valuable at all’ to ‘Very valuable’. For questions regarding the technology used in care, multiple answers could be selected from a list of technologies, or patients could report a technology that was not on the list by selecting ‘other’. One question provided a list of seven outcome measures for home-monitoring, of which patients had to rank a top three of the most valuable outcome measures. Furthermore, patients could indicate their preferred maximal number (from ‘0’ to ‘7’) and frequency (from ‘Daily’ to ‘Quarterly or less’) of home assessments. The complete survey is provided in Appendix 1.

### Patient recruitment and procedures

For the Netherlands, the national ALS registry was used. A subset of 375 patients who had given prior informed consent to be approached for future research were invited to participate. Patients received an invitation to participate either via e-mail or post, depending on whether an email address was available in the database. The e-mail included a link to an online platform (EDC Castor) with access to the patient information sheet, consent form, and survey. The same documents on paper, together with a postage paid envelope were sent by post to those who did not have an email address available. A reminder was sent by e-mail four weeks later to those who had not replied. Those who had not opened the email within four weeks, received the survey by post. In the UK, 221 patients who attended the Sheffield MND clinic were invited to participate, which included both patients living in Sheffield and the surrounding counties, as well as out of area patients who attended the Sheffield MND clinic for a second opinion or participation in clinical trials. Patients were invited to participate by postal mail and received a patient information sheet, consent form, a postage paid envelope and the survey. A reminder was sent after one month to those who had not replied. In Australia, a subset of 151 patients from Queensland, Victoria or Western Australia, who were listed in a national MND registry and previously consented to be contacted for future research were invited to participate. National calls for research participation were also distributed by social media by the Motor Neurone Disease Research Australia and FightMND foundations. Patients were sent an e-mail outlining the project; information included a patient information sheet and consent form, and instructions on how to contact study personnel. Upon completion of the consent form, patients were e-mailed a unique survey token, which provided access to the survey using an online platform (LimeSurvey). Reminder emails were sent to all consenting participants one month after consent, for patients who did not complete the survey.

### Statistical analysis

For statistical analysis, all 5-point Likert scales were converted to a 3-point scale (Disagree (1–2), Neutral (3) and Agree (4–5); Not valuable (1–2), Neutral (3) and Valuable (4–5)), and reported as the percentage of patients who selected the response. A chi-square test was used to assess differences in item responses and other nominal variables between the three countries, and a one-way ANOVA together with a Bonferroni post-hoc test were used to assess differences in continuous variables. Multinomial regression was used to assess whether survey items were related to covariates, i.e., age (younger adults < 65 years; older adults ≥ 65 years), sex (male; female) and site of disease onset (spinal; bulbar). One item required respondents to rank three of the seven proposed home measures for home-monitoring from most valuable to least valuable. Rank 1 received a score of 3, rank 2 a score of 2 and rank 3 a score of 1; measures not listed in the top 3 received a score of 0. The total rank score of each home measure was subsequently calculated by taking the sum of the rank scores, and were divided by the maximal possible score (all patients ranked a measure as 1st), resulting in a score between 0 and 1. For the comparisons between countries, the total rank scores of each home measure per country were normalized by dividing them by the highest possible score of that country.

## Results

In total, 332 patients with MND participated in the study; 200 in The Netherlands, 91 in the UK and 41 in Australia. The response rate for those directly contacted was 53.4% for The Netherlands, 41.6% for the UK, 27.1% for Australia; patient characteristics per country are presented in Table 1. Overall, the majority of patients had access to internet (95.6%) and used a digital device (smartphone, computer or tablet) several times per week (93.1%); this was similar across countries (*p* = 0.46, Table 1). Most patients (85.9%) had experience with the use of digital technology during care, such as electronic health records, mobile health apps, wearables, video consultations, email or mobile text messaging. An increased use of technology in healthcare due to the COVID-19 pandemic was reported by 48.6% of patients. In this subgroup of patients, 75.1% started using video consultations, 30.6% e-mail, 19.1% text messaging and 12.7% a mobile health app to receive care remotely.

**Table 1:** Patient characteristics at enrolment

Characteristic^a^	The Netherlands (*N* = 200)	United Kingdom (*N* = 91)	Australia (*N* = 41)	*p* value^c^
Sex, male (%)	135 (68.2)	55 (61.1)	29 (70.7)	0.42
Age at enrolment, years	63.4 (10.2)*	66.8 (10.2)*	64.8 (7.8)	0.029
Symptom onset, bulbar (%)	34 (17.2)	19 (21.3)	4 (12.1)	0.44
Symptom duration,^b^ months	42.1 (21.7–68.5)*	64.1 (27.1–148.3)*^†^	44.0 (24.6–77.7)^†^	< 0.001
Diagnostic delay,^b^ months	13.7 (6.9–29)	17.5 (8.4–29.5)	16.0 (5.3–25.5)	0.51
Method of completing questionnaire, digitally	171 (85.5)*	0 (0)*	100 (0)*	< 0.001
Current digital technology use, (> 1 times per week) *n* (%)				
Smartphone	169 (85.8)*	67 (79.3)*^†^	38 (92.7)^†^	0.017
Computer/laptop	123 (62.4)*	42 (50.6)*^†^	32 (78.0)^†^	0.003
Tablet	102 (51.8)*	53 (63.2)	31 (75.6)*	0.016
At least one of the above	188 (95.4)	83 (90.1)	39 (95.1)	0.46
Participated in research including at least one clinic visit, *n* (%)	90 (45.7)*	47 (52.2)^†^	29 (70.7)*^†^	0.024

Data are given in mean (standard deviation) or *n* (%). ^a^Due to missing values the number of responses (n) may differ per variable, ^b^data are median (25th–75th percentile), ^c^*p* value for group comparisons, *^†^significantly different between countries (*p* < 0.05)

### Attitude and preferences regarding home-monitoring

The majority of patients liked the idea of monitoring their own health at home (68.9%), although there were differences across the countries (UK = 58.8%, The Netherlands = 74.0%, Australia = 70.7%; *p* = 0.022). Of all patients, 14.6% indicated that they would not wish to perform measurements at home, which was similar across countries (*p* = 0.82). In the Netherlands, albeit a minority, women and older adults were more likely to dislike the idea of performing measurements at home, compared to men (21.3% vs 10.9%, *p* = 0.032) and younger adults (19.0% vs 10.4%, *p* = 0.017). There were no significant differences in attitude between patients with a spinal or bulbar symptom onset in any of the countries.

**Fig. 1:**
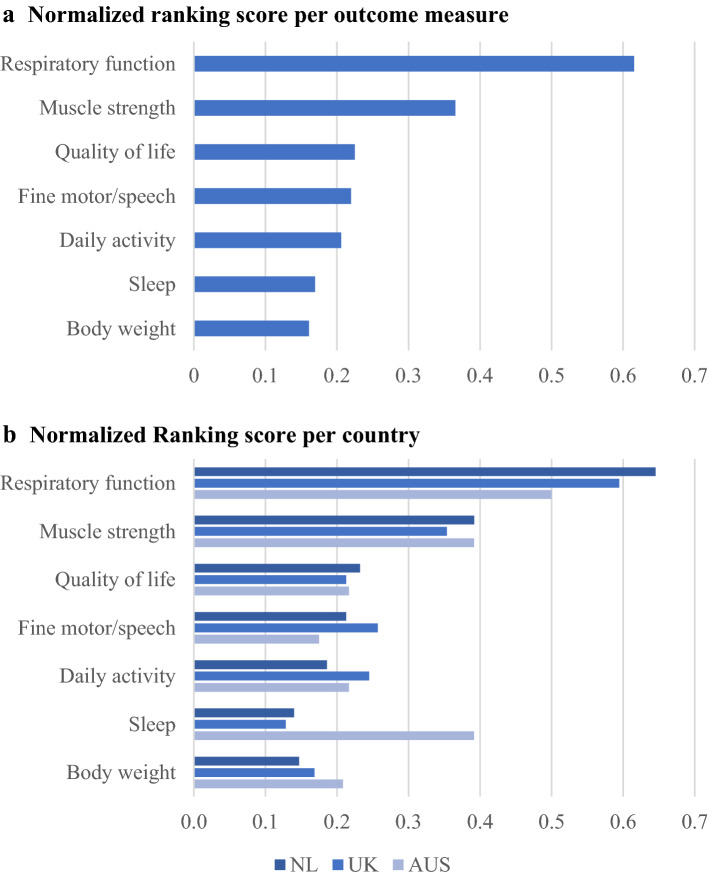
Most valuable outcome measure for home-monitoring according to patients. *NL* The Netherlands, *UK* United Kingdom, *AUS* Australia. Patients ranked a top 3 out of 7 proposed outcome measures from most valuable to least valuable. 1st place received a score of 3, 2nd place a score of 2, and 3rd a score of 1. *The ranking score is the sum of scores per outcome measure. **a** Ranking scores were normalized by dividing a ranking score by the highest possible ranking score, **b** Ranking scores were normalized per country by dividing a ranking score by the maximal possible ranking score per country

**Fig. 2:**
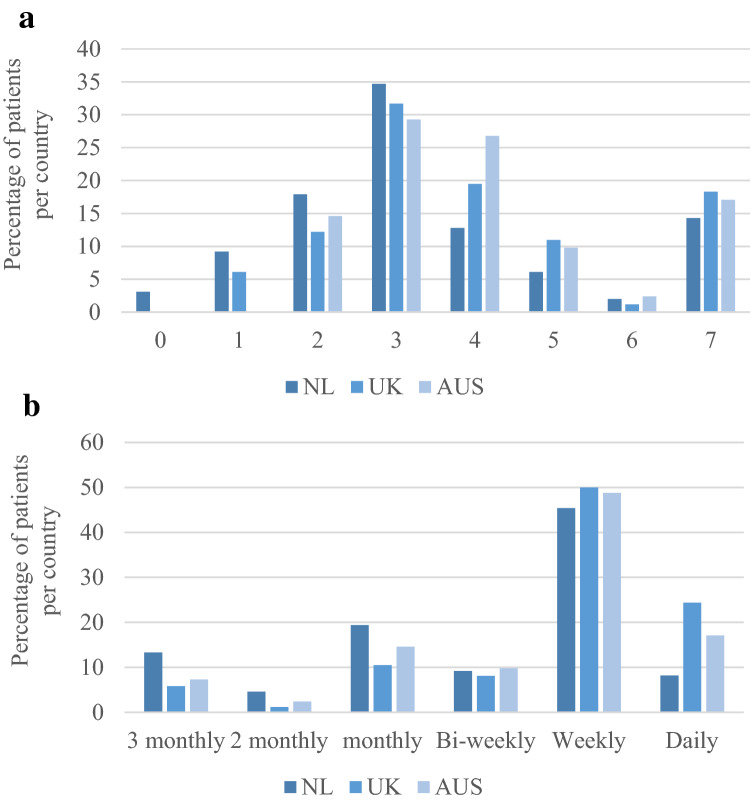
Preferred **a** maximum number of apps/devices and **b** frequency for home-monitoring according to patients. *NL* The Netherlands, *UK* United Kingdom, *AUS* Australia. Figures show the percentage of patients per country who chose **a** the number of apps/devices and **b** frequency for home-monitoring

The two outcome measures that were considered most valuable by patients for home-monitoring were respiratory function and muscle strength; the ranking of the other measures is presented in Fig. 1. Across all countries, 74.9% of patients were willing to use 3 or more devices for home-monitoring, and 60.7% of patients were willing to perform home-measurements at least weekly, and 86.1% of patients at least monthly (Fig. 2). Men across all countries were more likely to choose three or more devices for home-monitoring compared to women (80.3 vs 63.5%, *p* = 0.044); there were no significant differences in the number of devices or monitoring frequency preferences between age groups or patients with a spinal and bulbar symptom onset in any of the countries.

### Concerns with home-monitoring

**Fig. 3:**
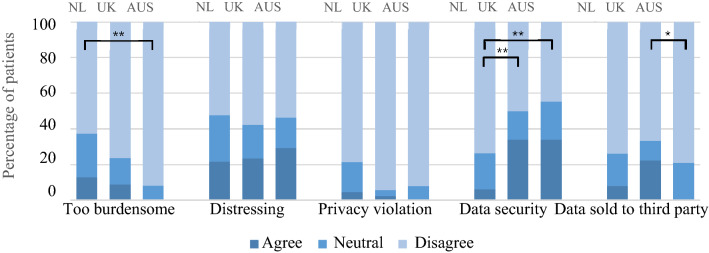
Concerns with home-monitoring per country. *NL* The Netherlands, *UK* United Kingdom, *AUS* Australia, *p < 0.05, **p < 0.001. Distressing = Feelings of distress due to self-monitoring of disease progression

**Fig. 4:**
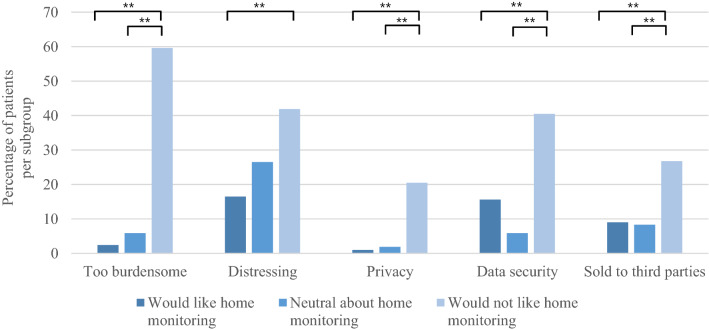
Concerns with home-monitoring based on patients’ attitude towards home-monitoring. We distinguished three subgroups: patients who would like home-monitoring (*n* = 221), patients who would not like home-monitoring (*n* = 47), and patients who are neutral about home-monitoring (*n* = 54). *p < 0.05, **p < 0.001. Distressing = Feelings of distress due to self-monitoring of disease progression

Some patients considered that home-monitoring would be too distressing (22.2%) or too burdensome (10.5%), and lead to problems with data security (16.8%), data being sold to third parties (11.0%), or privacy-related concerns (3.3%) (Fig. 3). Differences between countries were found in ‘home-monitoring being too burdensome’ between The Netherlands (13%) and Australia (0%, *p* = 0.001), in ‘data security’ between The Netherlands (6.2%) and the UK (34.1%, *p* < 0.001) and Australia (34.2%, *p* < 0.001), and in ‘data being sold to third parties’ between the UK (22.2%, *p* = 0.002) and The Netherlands (8%) and Australia (0%) (Fig. 3). Patients who would not like home-monitoring reported more concerns, compared to those who were neutral about or would like home-monitoring (Fig. 4). Furthermore, in the Netherlands, older adults (≥ 65 years) were more likely to think that home-monitoring would be too burdensome compared to younger adults (19.5 vs 8.4%, *p* = 0.008); there were no significant differences in concerns between males and females, or between patients with a spinal and bulbar symptom onset in any of the countries.

### Remote MND healthcare

Most patients (74.5%) would like to be remotely monitored by their multidisciplinary care team; this was similar across countries (*p* = 0.60). 10.1% of patients did not feel the need for remote care, with significantly more patients in The Netherlands (12.3%, *p* = 0.019) and Australia (14.6%, *p* = 0.001) reporting that they do not feel the need for remote care, compared to the UK (5.8%). Older adults were more likely to be reluctant towards remote care, compared to younger adults (13.5 vs 6.7%, *p* = 0.021); there was no difference in reluctance towards remote care between sexes or between patients with spinal *vs.* bulbar symptom onset in any of the countries. The potential benefits of digital healthcare technology that were valued most by patients were (1) improved communication with the multidisciplinary care team (75.9%) and (2) better insight into their disease course (83.0%).

### Remote participation in clinical research and trials

**Fig. 5:**
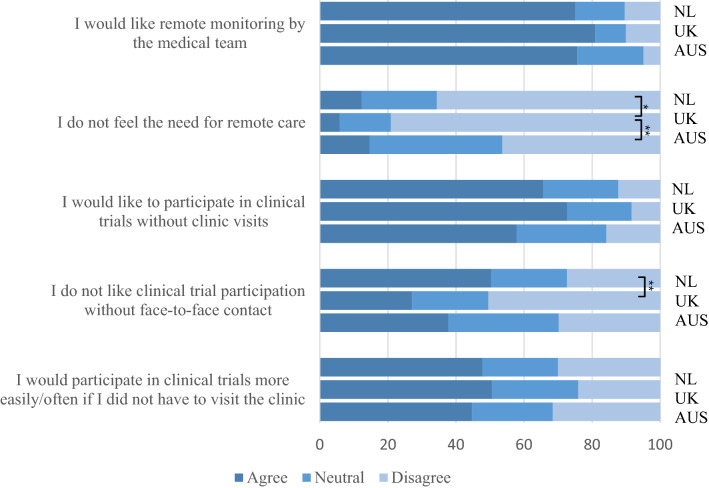
Patients’ attitude towards remote healthcare and remote clinical trials. *NL* The Netherlands, *UK* United Kingdom, *AUS* Australia, *p < 0.05, **p < 0.001

Approximately half of patients (50.6%) had participated in clinical research that required an in-clinic visit. In the other half of patients, the most common reasons for not participating were: (1) not having received an invitation to participate (44.0%), (2) thinking participation would be too burdensome (21.0%) and (3) travel distances being too far (19.0%). Out of all respondents, 65.2% liked the idea of participating in clinical trials without visits to the clinic, and 46.2% would participate in clinical trials more often/easily if this could be done remotely (Fig. 5). However, 41.0% of patients disliked the idea of participating in a clinical trial without personal contact with a healthcare professional. Respondents mostly valued the potential of digital healthcare technology to make clinical trials shorter (73.2%), and more accessible to a broader group of patients (82.7%).

## Discussion

We have shown that the majority of patients with MND in our cohort from The Netherlands, UK and Australia have a positive attitude toward performing MND-related measurements at home, remote monitoring by the multidisciplinary care team, and remote participation in clinical trials. Respiratory function and muscle strength were considered to be the most valuable measures for home-monitoring, and using three to four apps/devices at a weekly frequency was considered acceptable for home-monitoring. Our findings highlight that a subgroup of patients have concerns with the use of digital technology at home. Important concerns that need to be considered are patient burden and distress of being confronted with physical deterioration, and data security or use of data by third-party collaborators.

Previous studies on the use of remote digital technology in MND care have shown that self-monitoring of health-related outcomes and frequent communication with a multidisciplinary care team may improve the patients’ understanding of, and control over the disease, and enhance the continuity of care throughout the course of the disease [7, 8, 26–28]. Interestingly, the use of digital technology in MND care in our cohort is considerably higher compared to a UK cohort of patients with MND in 2015, despite the use of digital devices (e.g. laptop, computer, tablet) in daily life being similar [29]. This increase in digital technology use in care is likely affected by the COVID-19 pandemic. In turn, the increased diffusion of remote digital technology in clinical care may facilitate the decentralization of clinical trials and new digital efficacy endpoints may increase a trial’s ability to detect treatment benefit and help to accelerate clinical development [10, 30, 31]. On top of that, the use of remote digital technology may increase clinical trial enrolment since more patients would be willing to participate in clinical trials. The use of remote digital technology may also help to include a broader group of patients, such as those who are rapidly progressing or more severely disabled. As a result, the diversification of trial populations can potentially improve the generalizability of clinical trial results [32]. We should be aware of the risk; however, that patients who are compliant with using digital technology may be similar to the subset of patients who are already participating in clinical trials (e.g. male, slow disease progression, younger) [33].

Though the majority of patients had little concerns, a subgroup of patients were reluctant towards home-monitoring and the remote provision of care. Albeit a minority, women and older adults in The Netherlands were more likely to be reluctant compared to men and younger adults, which corresponds to previous research which found that women and older adults were less positive towards health technology and experienced more barriers with technology use, due to, among others, inexperience and lower self-efficacy with technology [34, 35]. Compared to The Netherlands, fewer patients from Australia had concerns regarding the burden of home-monitoring. This observation was mainly driven by older patients in The Netherlands who had the most concerns about digital technology. The difference with the Australian cohort could be due to selection bias (response rate of 27.1%), which may have resulted in a sample of patients with a positive bias towards digital technology use. Interestingly, concerns regarding data security were lower in The Netherlands compared to the UK and Australia. Based on existing literature this may be due to differences in interpretation and understanding of the term ‘data security’, as a result of differences in education level and familiarity with the terminology [36, 37]. These findings suggest that it is important to involve patients from various demographics at the design stage when determining how to measure remote digital health outcomes in care or clinical trials. By doing so, it will enhance familiarity with digital technology among people with lower digital literacy, help to select patient-friendly devices and assessments, and ensure that home monitoring is compliant for a broad group of patients.

Out of all remote digital health outcomes that were proposed in the present study, patients considered respiratory function and muscle strength to be the most valuable. These two outcome measures can provide patients with more insight into their disease (progression), and in turn, help patients make a decision on when to initiate non-invasive ventilation or start using assistive devices [38]. In addition, respiratory function and muscle strength are known to be related to disease progression, functional ability and quality of life in patients with MND [39–43], and are, therefore, important outcomes in both care and clinical trials. So far, direct assessments of muscle strength (e.g. grip strength and leg extension strength), and indirect assessments (e.g. plasma creatinine) have the potential to be used for home monitoring of muscle strength [9, 44–46]. For the home-monitoring of respiratory function, the assessment of vital capacity, maximal inspiratory pressure and patient-reported symptoms of dyspnea have been proposed in previous studies [47–51]. Future studies could focus on how respiratory function, muscle strength and other relevant digital health outcomes, such as cognitive impairment, can be best measured and utilized at home, with the involvement of patients with MND.

### Limitations

Strengths of the present study are the multi-centre design and inclusion of a cohort of patients with MND from different national backgrounds. A limitation is the potential of recruitment bias, since the average response rate was relatively low. In addition, a large portion of patients were recruited digitally, which increases the likelihood of a positive attitude towards digital technology use, or familiarity with technology in daily life. As such, it is likely that we missed patients with lower digital literacy or social economic status, since these populations experience more difficulties with gaining access to digital health technology, and participation in research [52, 53]. Despite this, there were only minor differences between digitally recruited patients and those recruited by postal mail. Nevertheless, it remains important to evaluate strategies to better involve MND populations with lower digital literacy and social economic status, and facilitate their engagement in research. Furthermore, we did not assess cognitive impairment, which is common in patients with MND and could have affected the patient’s ability to indicate their preferences and concerns. It is likely that caregivers have assisted with filling in the survey in the present study. It may be important for future research to assess the level of cognitive impairment and whether patients were assisted by a caregiver, in order to better interpret the data.

## Conclusion

Patients with MND in The Netherlands, United Kingdom and Australia report a willingness to use digital healthcare technology for home-monitoring, and have a positive attitude towards receiving multidisciplinary care remotely and participating in decentralized clinical trials. Future studies should investigate how remote digital outcomes can be best utilized at home and implemented in daily MND care and clinical trials, preferably as a collaborative effort between patients and their caregivers, healthcare professionals and researchers.

## Supplementary Information

Below is the link to the electronic supplementary material.

Supplementary file1 (DOCX 74 kb)
