# Supplementary material for: Patient perspectives on digital healthcare technology in care and clinical trials for motor neuron disease: an international survey
Source: J Neurol. 2022 Jul 18;269(11):6003–13. doi: 10.1007/s00415-022-11273-x (PMC9294855; doi:10.1007/s00415-022-11273-x)

**Supplementary material 1**

**The use of digital technology in ALS/MND care and clinical trials**

The purpose of this questionnaire is to investigate what people with ALS/MND think of 1) the use of digital technology in care and clinical trials, 2) receiving care remotely, 3) participating in clinical trials remotely and 4) performing health assessments at home.

1. **The use of digital technology and internet**

The use of digital technology includes the use of e-mail, texting, smartphones, tablets, computers/laptops, mobile apps, wearables and other electronic devices that can measure variables, such as a Fitbit, smartwatch, Bluetooth scale or respiratory function test.

- 1. **I have internet access at home.**


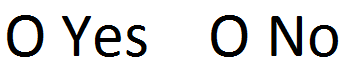


- 1. **I use a tablet.**


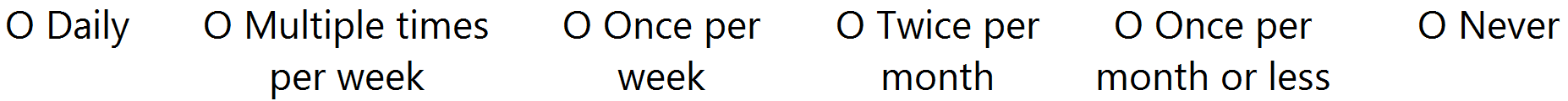


- 1. **I use a computer/laptop.**


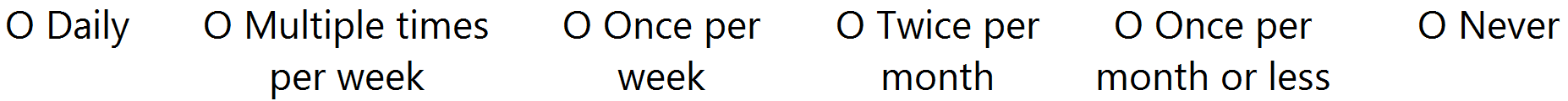


- 1. **I use a smartphone.**


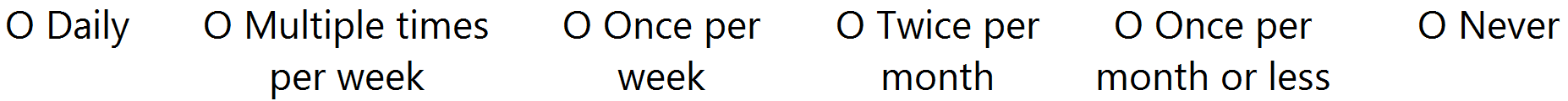


- 1. **I use internet (websites).**


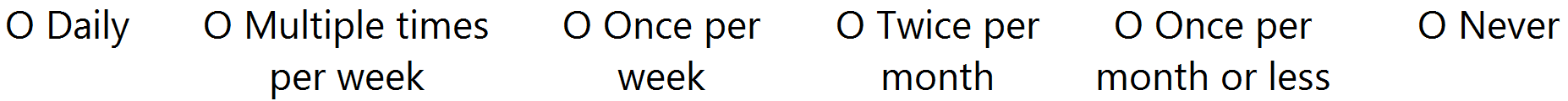


- 1. **I use e-mail.**


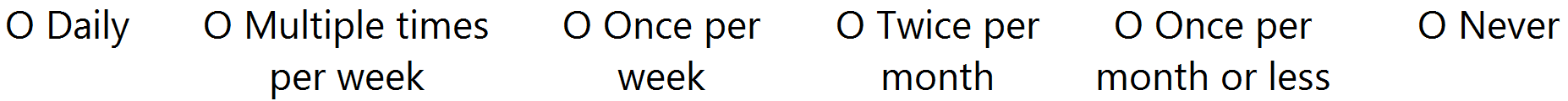


- 1. **Irrespective of your current impairments, do you experience difficulties understanding how to operate any of the above-mentioned technologies? (You may give multiple answers)**

□ Yes, a computer/laptop
□ Yes, a smartphone
□ Yes, a tablet
□ Yes, internet/websites
□ Yes, e-Mail
□ Yes, Texting/Whatsapp/iMessage
□ No/ none

- 1. **Which digital technologies have you used in care for communication with a healthcare professional or for assessing your health? (You may give multiple answers)**

□ e-Mail

□ Text messaging/Whatsapp/iMessage

□ Wearable (e.g. Smartwatch, Fitbit)

□ Mobile health app

□ Electronic health record/ digital patient environment

□ Video call/ videoconference (e.g. Skype, Zoom)

□ Other digital technology: __________________________________

□ None

- 1. **Has the use of digital technology in care changed since the start of the COVID-19 pandemic?**

O Yes, I use more digital technology

O Yes, I use less digital technology
O No, nothing changed

- 1. **Since the start of the COVID-19 pandemic, what digital technology that you did not yet use in care have you started using? (You may give multiple answers)**

□ e-Mail

□ Text messaging/Whatsapp/iMessage

□ Wearable (e.g. Smartwatch, Fitbit)

□ Mobile health app

□ Electronic health record/ digital patient environment

□ Video call/ videoconference (e.g. Skype, Zoom)

□ Other digital technology: __________________________________

□ None

1. **The remote provision of care**
   1. I would like to stay in contact with the medical team from home (in between clinic visits).


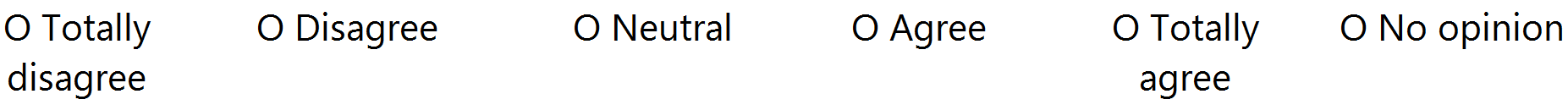


- 1. **I like the idea of the medical team remotely monitoring my health.**


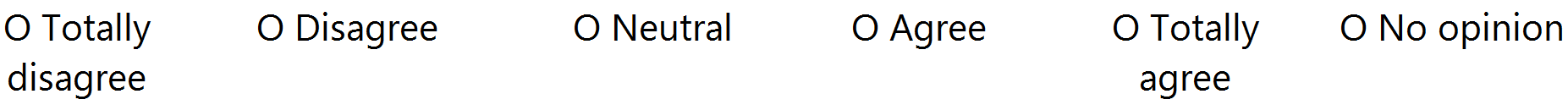


- 1. **I like the idea of monitoring my own health.**


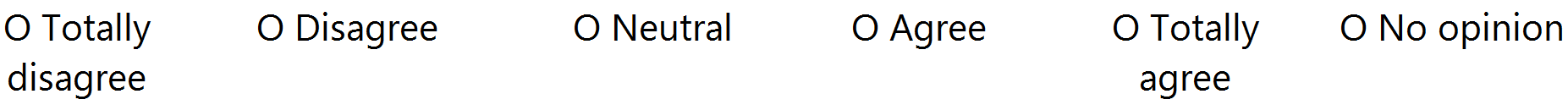


- 1. **I do not feel the need for receiving care remotely.**


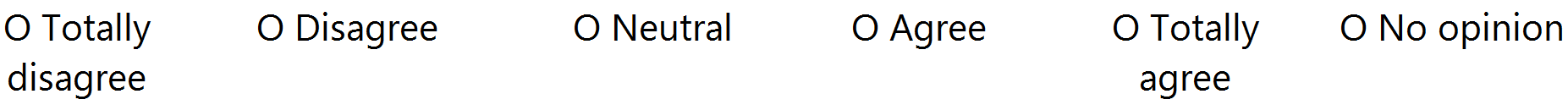


- 1. **I find it off-putting to monitor my own health.**


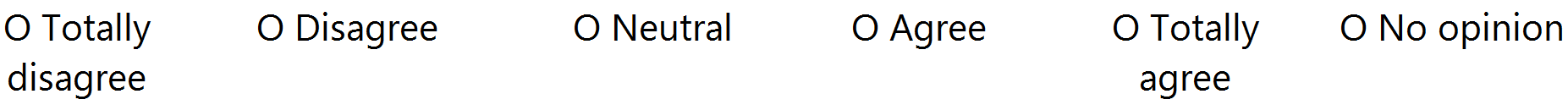


1. **Remote participation in clinical trials**
   1. **I have participated in a clinical trial for which I had to visit the clinic.**

O Yes
O No
O I don’t know

- - 1. **If not, what was the reason? (You may give multiple answers)**

□ Too burdensome
□ No time
□ No interest
□ Not invited
□ Not eligible
□ No way of transportation
□ No caregiver
□ Travel-related costs
□ Too far
□ Other

- 1. **I like the idea of participating in clinical trials or clinical research from home without clinic visits.**


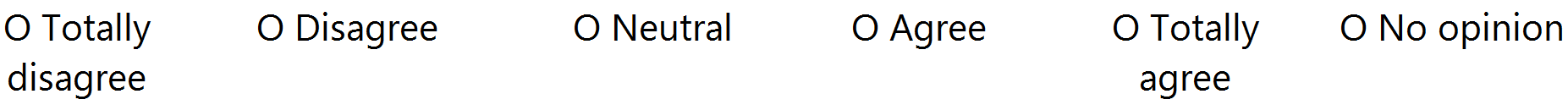


- 1. **I do not like participating in clinical trials or clinical research without face-to-face contact with a healthcare professional.**


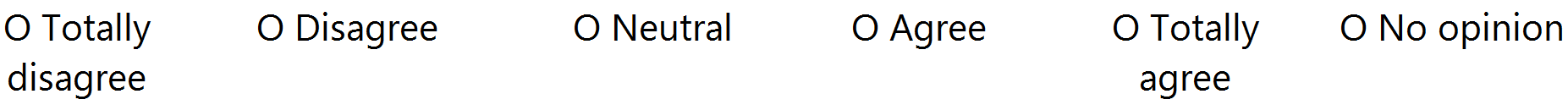


- 1. **I would participate in clinical trials or clinical research more easily/often if I did not have to visit the clinic.**


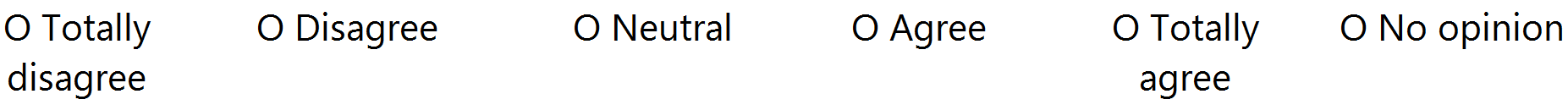


1. **Potential benefits**

The use of digital technology for self-monitoring at home can lead to number of changes in care and clinical trials. Please indicate how much you value each change.

- 1. **Fewer visits to the clinic.**


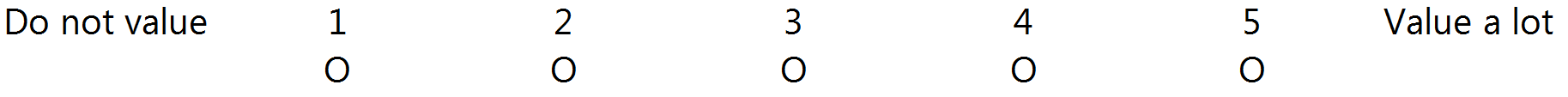


- 1. **Improved communication with medical team/ investigator.**


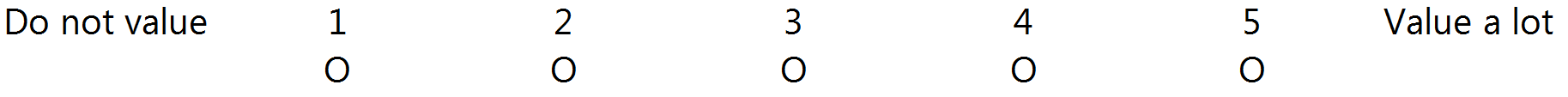


Care

- 1. **Reduced burden from clinic visits/ long clinic days.**


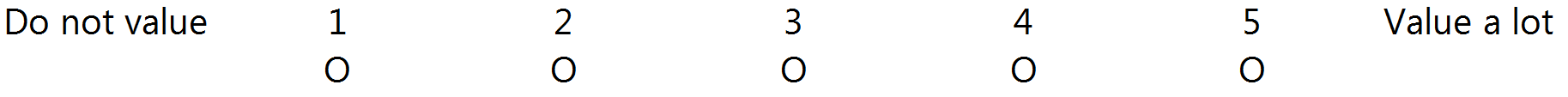


- 1. **Better timing of clinic/ hospital visits.**


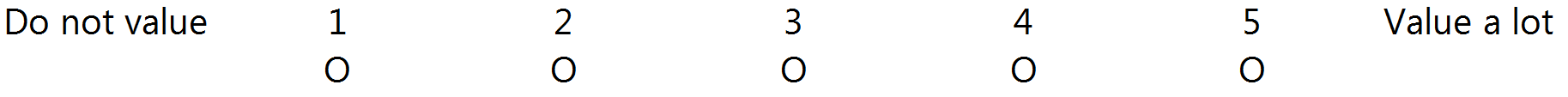


- 1. **Better timing of interventions and information in care.**


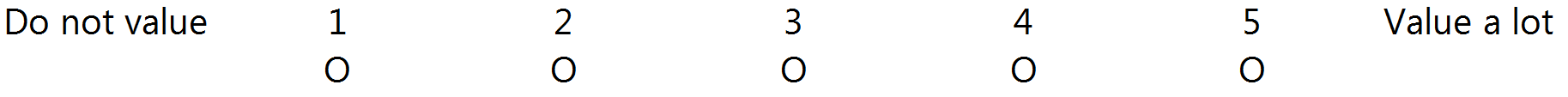


- 1. **Better timing of the provision of assistive devices in care.**


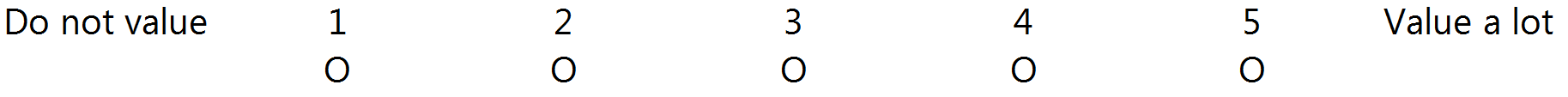


- 1. **Better insight in my disease course (better preparation for future).**


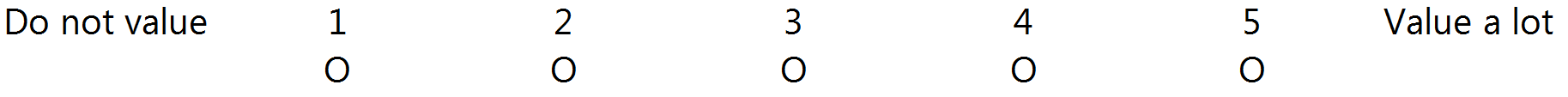


- 1. **Better insight in current health status/ functional status.**


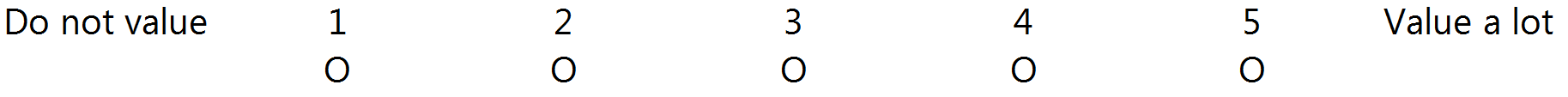


Clinical trials

- 1. **Reduced burden of clinical trial participation.**


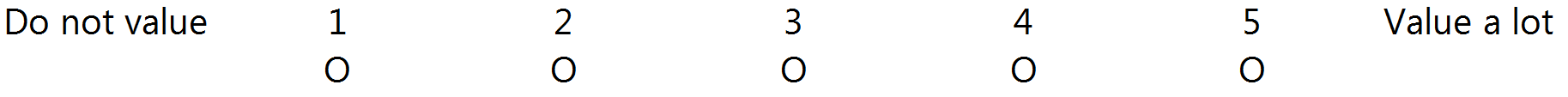


- 1. **Clinical trials become cheaper and faster.**


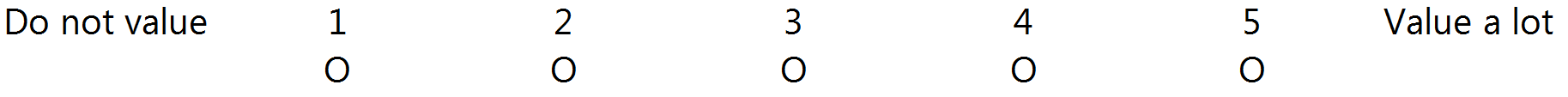


- 1. **Clinical trials become accessible for a broader group of patients.**


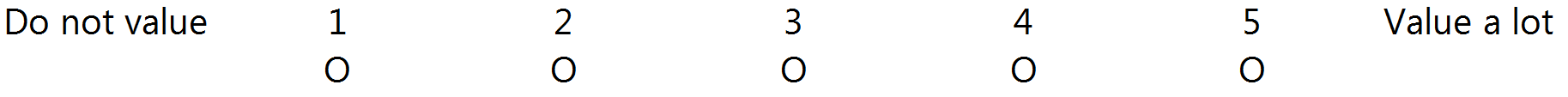


1. **What to measure?**

There are a number of proposed assessments that patients with ALS/MND could perform at home. Please indicate to what extent you believe the following assessments are valuable. If you are unable to perform one of the following assessments, please indicate how much you would have valued the assessment if you had been able to.

- 1. **Respiratory function testing.** This measure consists of performing three maximal efforts of in- and expiration using a portable pulmonary device.


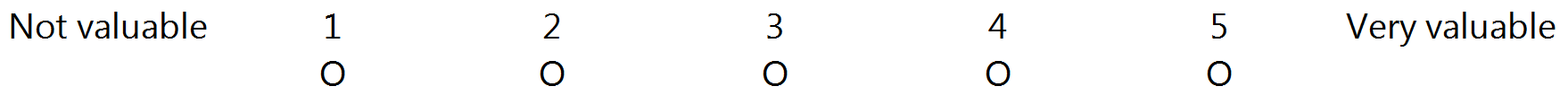


- 1. **Body weight.** This measure consists of using a body weight scale.


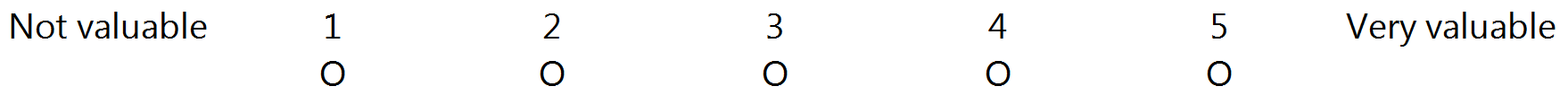


- 1. **Daily physical activity**. This measure consists of wearing a small wearable on your hip.


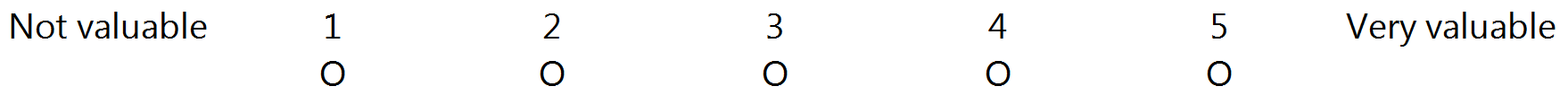


- 1. **Muscle strength.** This measure consists of performing three maximal efforts of a strength test.


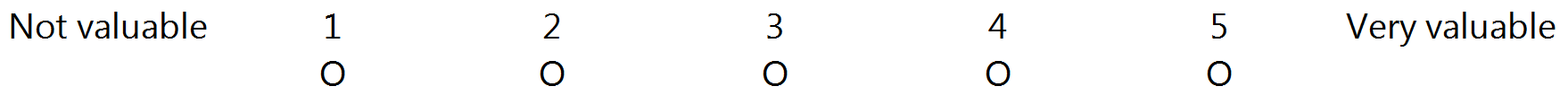


- 1. **Quality of life and symptoms.** This measure consists of filling out a digital questionnaire on an electronic device on quality of life and symptoms.


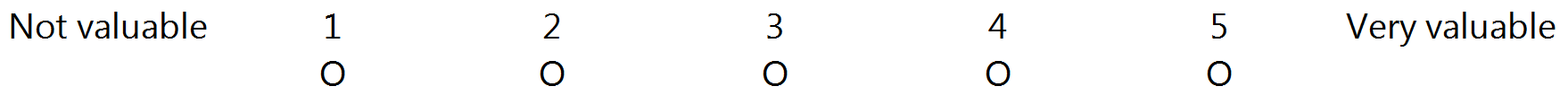


- 1. **Fine motor skills and speech function.** This measure consists of performing tasks on a smartphone/tablet for fine motor skills and speech function.


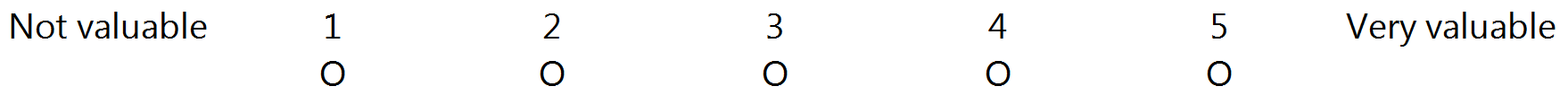


- 1. **Sleep.** This measure consists of a device that is placed under your mattress, that measures movement at night during sleep.


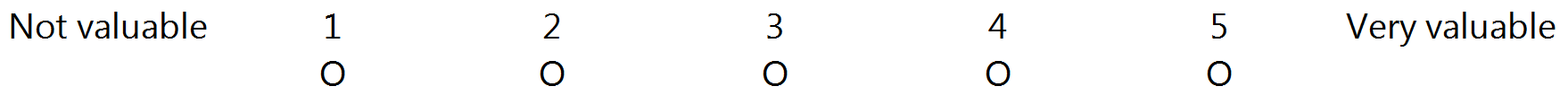


1. **Ranking**
   1. **Which of the before-mentioned assessments do you find most valuable?**

Give your top 3. From 1 (most valuable) to 3 (less valuable).
Fill in the number of the assessment (e.g. 5.3, 5.6, 5.1).

1. _____
2. _____
3. _____
4. **How much to measure.**
   1. **The maximal number of devices or assessments that I find acceptable to use at home is:**


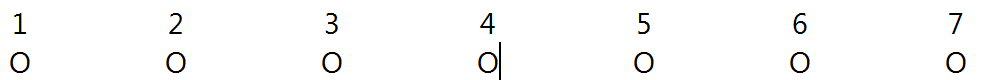


- 1. **The highest frequency that I find acceptable for performing assessments at home is:**


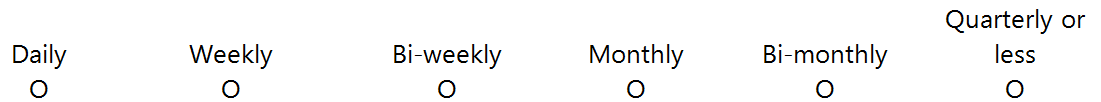


1. **Preferences and concerns**
   1. **I would like to see and access my own data.**


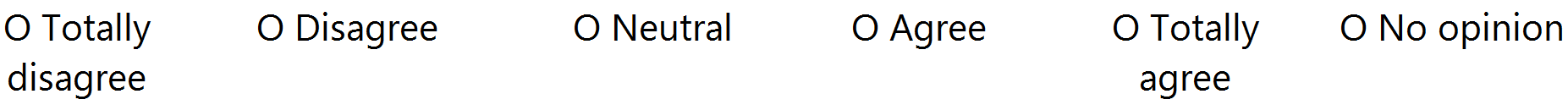


- 1. **I would like to have insight into my current rate of disease progression.**


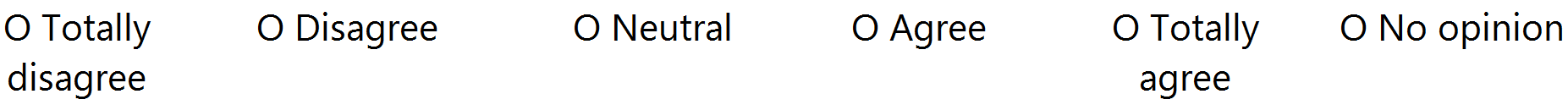


- 1. **I would like to have insight into my predicted disease course and/or prognosis.**


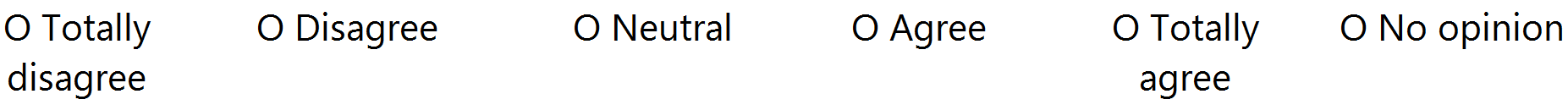


- 1. **I believe that the use of telehealth at home will be too burdensome.**


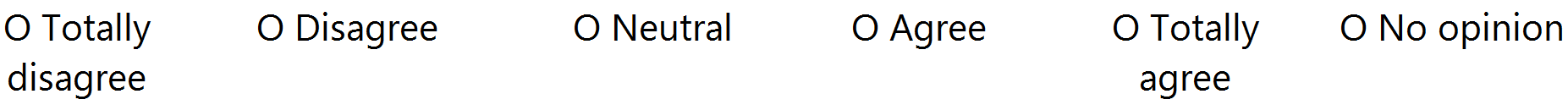


- 1. **I would not like to perform measurements at home.**


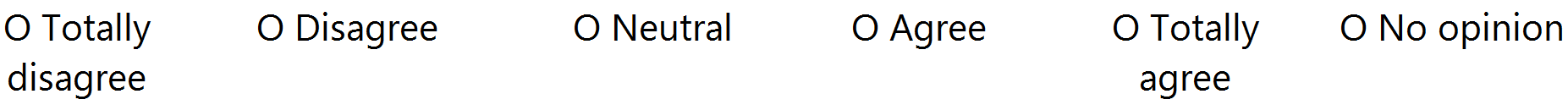


- 1. **I believe that the use of telehealth violates my privacy.**


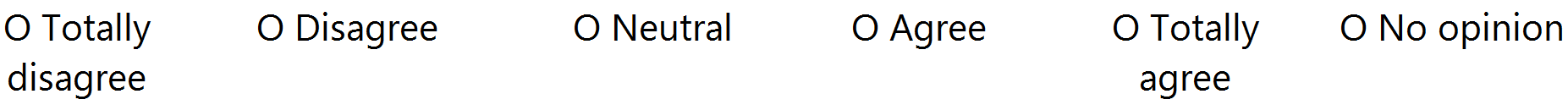


- 1. **I believe that data safety is an issue for telehealth.**


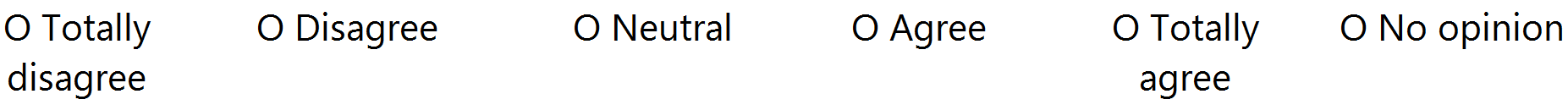


- 1. **I am afraid that my data will be used by or sold to third-parties**


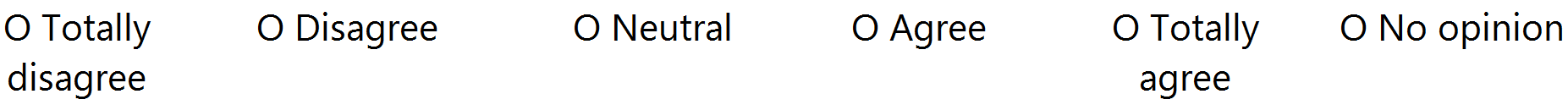

Supplement: Supplementary file 1 — Supplementary file1 (DOCX 74 kb) [file 415_2022_11273_MOESM1_ESM.docx]
